# hnRNP F Complexes with Tristetraprolin and Stimulates ARE-mRNA Decay

**DOI:** 10.1371/journal.pone.0100992

**Published:** 2014-06-30

**Authors:** Boris Reznik, Sandra L. Clement, Jens Lykke-Andersen

**Affiliations:** Division of Biological Sciences, University of California, San Diego, La Jolla, California, United States of America; McGill University, Canada

## Abstract

The tristetraprolin (TTP) family of zinc-finger proteins, TTP, BRF1 and BRF2, regulate the stability of a subset of mRNAs containing 3′UTR AU-rich elements (AREs), including mRNAs coding for cytokines, transcription factors, and proto-oncogenes. To better understand the mechanism by which TTP-family proteins control mRNA stability in mammalian cells, we aimed to identify TTP- and BRF1-interacting proteins as potential TTP-family co-factors. This revealed hnRNP F as a prominent interactor of TTP and BRF1. While TTP, BRF1 and hnRNP F are all RNA binding proteins (RBPs), the interaction of hnRNP F with TTP and BRF1 is independent of RNA. Depletion of hnRNP F impairs the decay of a subset of TTP-substrate ARE-mRNAs by a mechanism independent of the extent of hnRNP F binding to the mRNA. Taken together, these findings implicate hnRNP F as a co-factor in a subset of TTP/BRF-mediated mRNA decay and highlight the importance of RBP cooperativity in mRNA regulation.

## Introduction

Messenger RNA (mRNA) degradation plays a critical role in gene expression and cell metabolism by preventing overexpression of proteins and by recycling nucleotides back to the cellular pools. Tristetraprolin (TTP; also called Zfp36 and Tis11) is an RNA binding protein that promotes rapid decay of a subset of mRNAs containing AU-rich elements (AREs) in the 3′ untranslated region (UTR) [1]–[3]. While TTP does not appear to have catalytic mRNA decay activity of its own, it interacts with several components of the cellular mRNA decay machinery including deadenylases, decapping factors, and exonucleases, to activate decay of target mRNAs [4]–[7]. Two mammalian homologs of TTP, BRF1 (also called Zfp36L1 and Tis11b) and BRF2 (also called Zfp36L2 and Tis11d), appear to have similar RNA binding properties and decay activities as TTP [5], [8]–[11].

The post-transcriptional regulation of ARE-containing mRNAs is complex. Upwards of 8% of mammalian mRNAs have predicted AREs [12]. Many ARE mRNAs encode for highly regulated factors, including cytokines, growth factors, transcription factors, and early response genes [13], [14]. At least twenty confirmed and putative AU-rich element binding proteins (AUBPs) have been identified thus far [13]. The proper regulation of ARE mRNAs by AUBPs is important for homeostasis and normal physiology, and misregulation is often associated with detrimental effects to health and fitness. For example, TTP knockout mice display severe autoimmune pathologies and systemic inflammation that is attributable to increased levels of the cytokine tumor necrosis factor-α (TNFα) due to slower decay of its mRNA in macrophages from these animals [3], [15]. Although BRF1 and BRF2 knockout mice die at different stages of development [16]–[18], tissue-specific conditional double mutants develop leukemia and misregulate oncogenic transcription factor Notch1, an ARE-containing mRNA [11]. Most often, AUBPs target ARE mRNAs for degradation, but some AUBPs stabilize mRNAs or regulate translation [13]. For example, HuR, a member of the ELAV (embryonic lethal, abnormal vision) family proteins, which is expressed ubiquitously in most cell types, stabilizes many of the same ARE mRNAs that are targeted for decay by other AUBPs [19], [20]. How TTP and other AUBPs identify and regulate specific substrate mRNAs amidst all the ARE-containing mRNAs and other AUBPs in the cell is not well understood.

The best characterized targets of TTP are the mRNAs for the cytokines TNFα and GMCSF, which were identified in the initial studies of the TTP knockout mouse [3], [15], [21]. Many additional TTP substrate mRNAs have been discovered since [22]–[24], and studies of the tandem zinc finger RNA binding domain of TTP has demonstrated high binding affinity for the ARE nonameric sequence UUAUUUAUU [25]–[28]. While TTP has been shown to bind and regulate the decay of ARE mRNAs, there is evidence from global mRNA analyses to suggest that TTP may also regulate many non-ARE containing mRNAs. For example, only 23 of 250 stabilized mRNAs in fibroblasts derived from TTP knockout mice contain predicted TTP binding sites [24] and only ∼10% of the 400 TTP-associated mRNAs identified in human dendritic cells appeared to contain an ARE [23]. In contrasting studies, most of the 128 mRNAs associated with TTP in mouse macrophage cells contained the ARE pentamer sequence (AUUUA) [22], and 84% of mRNAs associated with exogenous TTP in HEK293 cells contained the UAUU sequence, the half-site of the preferred TTP ARE nonameric binding sequence [29]. Thus, it appears that TTP RNA binding is diverse and possibly not limited only to mRNAs containing AREs. Moreover, the profile of TTP-associated mRNAs may vary by tissue or cell-type. Additionally, not all TTP-bound mRNAs require TTP for degradation [22], demonstrating the complexity of TTP regulation of mRNA.

Given the abundance of predicted cellular ARE mRNAs, the potential competition for binding to these transcripts from other AUBPs, and the possibility that TTP associates with a large number of non-ARE mRNAs in the cell, much remains to be known about how TTP and its homologs BRF1 and BRF2 identifies and regulates target mRNAs. The objective of this study was to identify proteins that exist in complex with TTP and BRF1 and might serve as TTP/BRF co-factors. Two prominent interacting proteins were identified, hnRNP F and CAD. Given the importance of hnRNP F as an abundant component of messenger ribonucleoproteins (mRNPs) and previous implications of hnRNP F in mRNA regulation we focused our studies on this protein. We found that hnRNP F forms RNA-independent complexes with TTP and BRF1 and that depletion of hnRNP F impairs the decay of a subset of TTP mRNA substrates. Thus, hnRNP F is a co-factor in degradation of a subset of TTP/BRF1-target mRNAs.

## Results

### Identification of TTP and BRF1 interacting proteins

**Figure 1:**
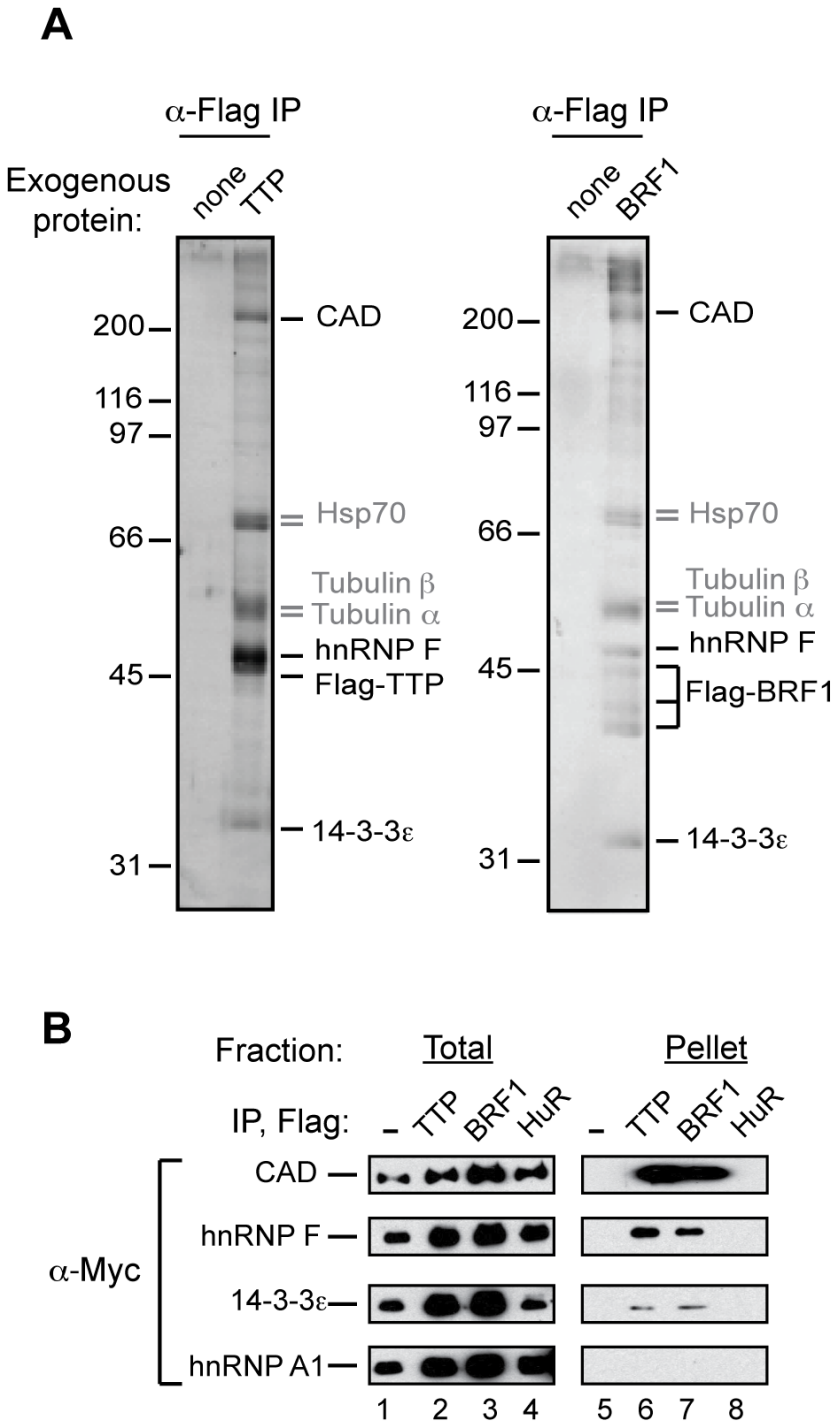
hnRNP F and CAD form RNA-independent complexes with TTP and BRF1. (A) Silver-stained SDS-polyacrylamide gels showing proteins that co-purify with stably expressed Flag-tagged TTP (left gel) or BRF1 (right gel) from extracts of T-REx HEK 293 cell lines after RNase-treatment. Proteins co-purified from cells expressing no Flag-tagged protein are run alongside as controls (lanes labeled ‘none’). (B) Western blots of anti-Flag IP reactions from RNase-treated extracts of HEK 293T cells transiently expressing Flag-tagged TTP (lane 6), BRF1 (lane 7), HuR (lane 8), or empty vector control (lane 5). Precipitates were probed for the presence of co-expressed Myc-tagged CAD, hnRNP F, 14-3-3ε, or hnRNP A1 as indicated. Lanes 1–4 shows 5% of the total extract for each IP reaction.

To gain mechanistic insight into the activity of TTP-family proteins in ARE-mRNA decay, we decided to isolate proteins that biochemically associate with TTP and BRF1. Human embryonic kidney (HEK) 293T cell lines were generated to stably express Flag-tagged TTP and BRF1 proteins under the control of a tetracycline inducible promoter to allow for titration of exogenous protein levels. Immunoprecipitation (IP) experiments were performed from RNase-treated extracts of cells expressing Flag-tagged TTP and BRF1 at levels similar to endogenous BRF1 (data not shown). Interacting proteins were separated by SDS-PAGE and visualized by silver staining. As seen in Figure 1A, TTP and BRF1 displayed remarkably similar profiles of co-purifying proteins with none of the co-purifying proteins observed from cells expressing no Flag-tagged proteins. Mass spectrometry analysis of individual gel bands identified 14-3-3ε, hnRNP F (heterogeneous nuclear ribonucleoprotein F), and CAD (carbamoyl-phosphate synthetase 2, aspartate transcarbamylase, and dihydroorotase) as prominent TTP and BRF1 interacting proteins (Figure 1A). In addition, Tubulin α, Tubulin β, and Hsp70 (heat shock protein 70) were identified, but these proteins were excluded from further study since we commonly find them co-purifying with Flag-tagged proteins [6]. The identification of 14-3-3ε, a previously characterized TTP-interacting protein [30], validated our experimental conditions.

To verify the identified protein interactions we performed co-IP assays with exogenously expressed, tagged proteins from RNase-treated HEK 293T extracts. As seen in Figure 1B, Myc-tagged CAD, hnRNP F and 14-3-3ε all co-purified with Flag-tagged TTP and BRF1, but not with Flag-tagged HuR, an ARE-mRNA stabilizing protein that served as a negative control (compare lanes 6, 7 to lane 8). An additional negative control, the RNA binding protein hnRNP A1, did not co-IP with either TTP or BRF1 (bottom panel). Thus, we confirmed that exogenously expressed CAD, hnRNP F and 14-3-3ε exist in RNA-independent complexes with TTP and BRF1.

### hnRNP F complexes with TTP in stimulated macrophage cells

hnRNP F is an RNA binding protein belonging to the hnRNP superfamily and has previously been implicated in multiple steps of mRNA metabolism and gene expression regulation [31], [32], including repression of translation in *D. melanogaster* [33] and translation regulation of neuronal mRNAs [34]. Given the overlap between known functions of TTP-family proteins and hnRNP F, we focused on hnRNP F for the remainder of this study; the potential role of CAD in TTP/BRF1 function is a topic for future studies.

**Figure 2:**
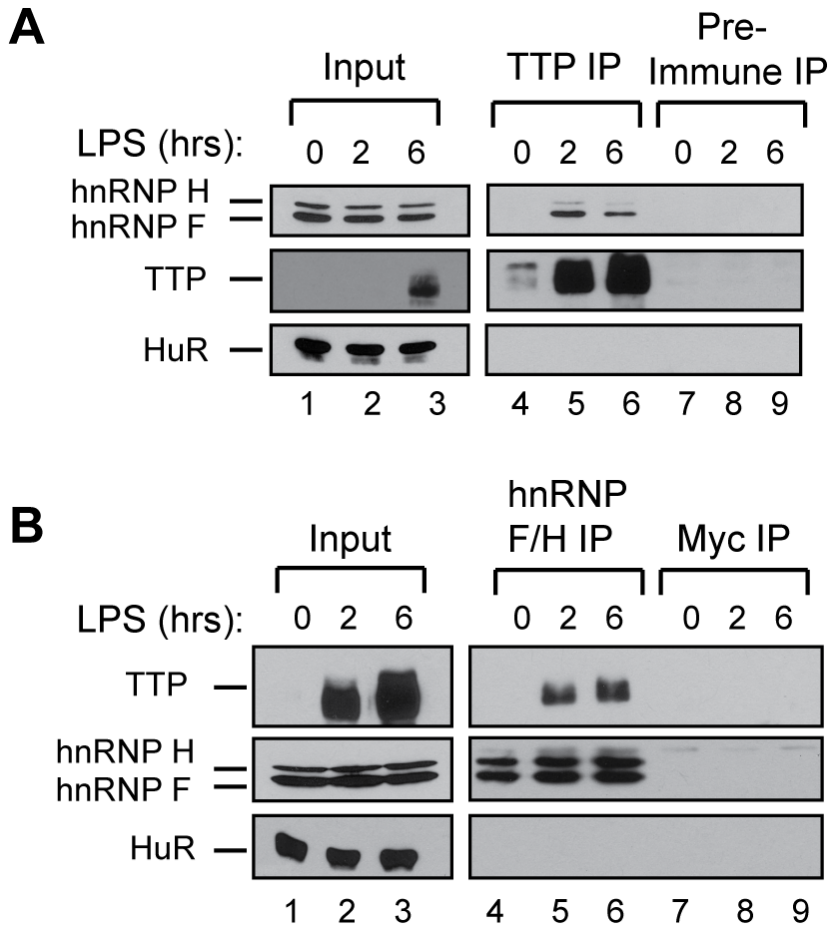
hnRNP F complexes with TTP/BRF1 in LPS-stimulated RAW macrophages. (A) Western blots for endogenous proteins that co-IP with anti-TTP (lanes 4–6) or pre-immune sera (lanes 7–9) from RNase-treated extracts of RAW264.7 cells stimulated with 100 ng/ml LPS for the indicated times. Precipitates are compared with 1% of the input (lanes 1–3). The anti-hnRNP F/H antibody recognizes both hnRNP F (bottom band) and hnRNP H proteins (top band). (B) Same as panel *A,* except the IP was performed with anti-hnRNP F/H (lanes 4–6) or anti-Myc (lanes 7–9).

To test whether endogenous TTP and hnRNP F associate in a biochemical complex, we took advantage of the mouse macrophage cell line, RAW264.7 (hereafter called RAW), in which TTP expression is induced upon treatment with lipopolysaccharide (LPS) (Figures 2A and B, left panels). As seen in the co-IP experiments in Figures 2A and 2B, endogenous hnRNP F can be detected to co-purify with TTP when TTP is induced after 2 and 6 hours of LPS treatment, but not prior to LPS treatment when TTP is expressed only at low levels (compare lanes 5, 6 with 4). The fraction of total cellular TTP that exists in complex with hnRNP F appears to decrease over the time course (compare hnRNP F in lanes 6 and 5 in Figure 2A, and TTP in lanes 6 and 5 versus 3 and 2 in Figure 2B). The hnRNP F antibody also detected hnRNP H1 and/or hnRNP H2, two homologous proteins that share 96% amino acid identity with each other and 70% amino acid identity with hnRNP F [35]. hnRNP H protein(s) (we could not distinguish between hnRNP H1 or hnRNP H2) likewise co-purify with TTP from RAW cells (Figure 2A, lanes 5 and 6). By contrast, the negative control RNA binding protein HuR did not co-purify with TTP (lower panels), and hnRNP F and TTP did not co-purify with non-specific antisera (Figures 2A and 2B, lanes 7–9). Thus, TTP can be observed in an RNA-independent complex with hnRNP F, and at least one of its close hnRNP H homologs, under conditions in which TTP is induced in a macrophage cell line. The absence of other tested RNA binding proteins from the TTP/BRF1-hnRNP F/H complexes (Figures 1 and 2) validates that these are RNA-independent interactions, although it cannot be ruled out that interactions are bridged by additional proteins.

### hnRNP F stimulates decay of an ARE mRNA that contains multiple hnRNP F binding sites

**Figure 3:**
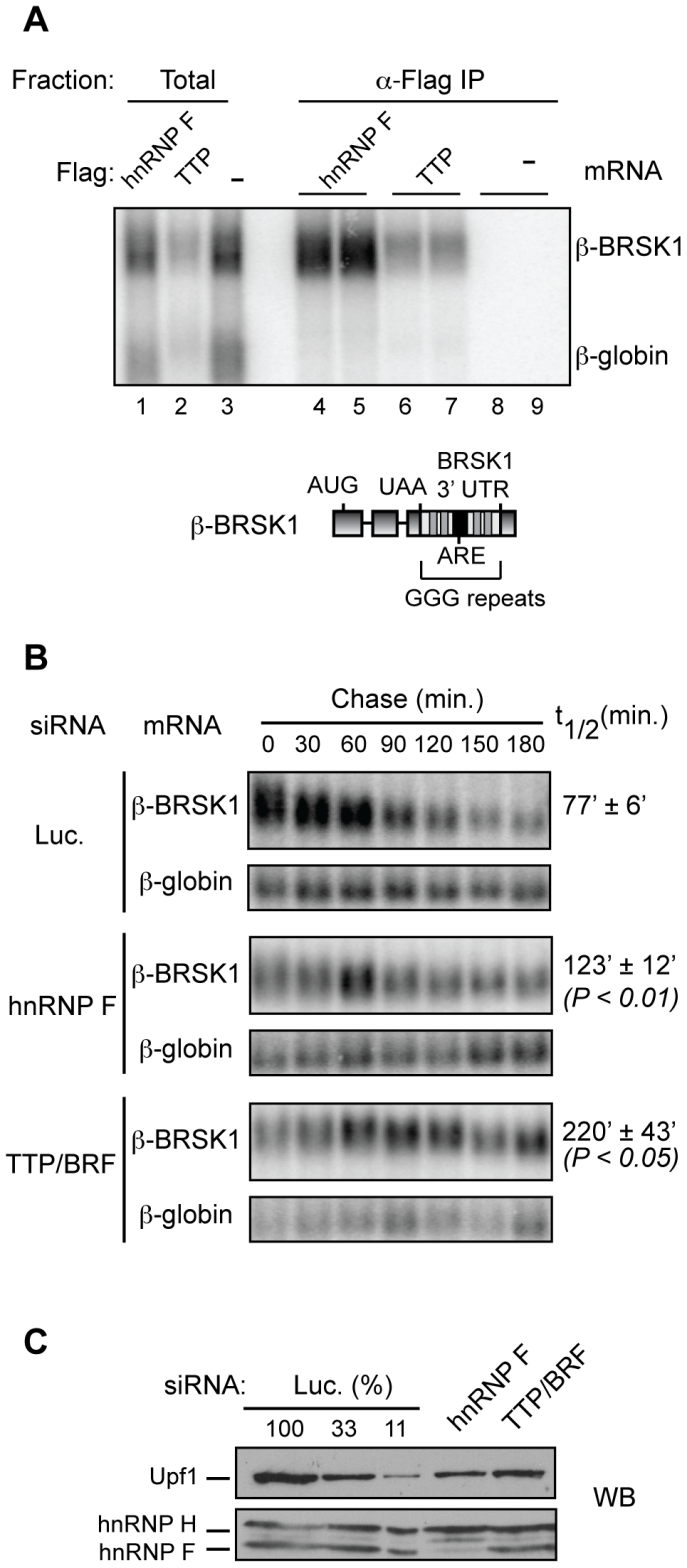
hnRNP F binds to and stimulates the decay mediated by the BRSK1 ARE-containing 3′UTR. (A) Northern blot showing reporter mRNAs that co-precipitate with transiently expressed Flag-tagged hnRNP F (lanes 4, 5), TTP (lanes 6, 7), or empty vector (lanes 8, 9) in HEK 293T cells. Precipitates and 5% of total extract (lanes 1–3) were probed for the presence of β-globin and β-BRSK1 mRNAs. Bottom, A cartoon schematic of the β-BRSK1 mRNA reporter with the 3′UTR from BRSK1 gene containing an ARE (black bar) and G-rich sequence repeats (grey bars) downstream of the β-globin coding region. (B) Northern blots showing mRNA decay of the β-BRSK1 mRNA reporter in HeLa Tet-off cells transfected with siRNAs targeting hnRNP F, TTP/BRF1/BRF2 (TTP/BRF), or luciferase (Luc.) as indicated. Numbers above lanes refer to times in minutes after transcriptional shut-off by tetracycline addition (Chase). Levels of the reporter mRNA was normalized to the constitutively expressed β-globin control mRNA and the average half-life (t_1/2_) and the standard error of the means was determined from 6 independent experiments. P-value was calculated with Students t-test (paired two-tailed). (C) HeLa Tet-Off cells were transfected with the corresponding siRNAs and knockdown of hnRNP F was assessed by Western Blot. Upf1 served as a loading control. Numbers above lanes with Luc siRNA samples refer to the percent of extract loaded.

To investigate whether hnRNP F affects TTP function, we first tested the importance of hnRNP F for the degradation of an mRNA substrate with multiple predicted binding sites for both hnRNP F and TTP. In vitro binding studies have identified the sequence DGGGD (where D is A, G or U) as a consensus binding site for hnRNP F and hnRNP H proteins [36]. The 494-nucleotide 3′UTR of BRSK1 mRNA contains a 37-nucleotide ARE with two nonameric predicted high affinity TTP binding sites (UUAUUUAUU; and a total of three AUUUA sequences) flanked upstream and downstream by thirteen DGGGD hnRNP F/H consensus sites. The 3′ UTR from the BRSK1 mRNA was inserted into a β-globin reporter mRNA, replacing the existing 3′UTR. The RNA-IP assays shown in Figure 3A verified that hnRNP F and TTP both assemble with the BRSK1 3′UTR, as the β-BRSK1 mRNA is enriched with both Flag-hnRNP F (lanes 4 and 5) and Flag-TTP (lanes 6 and 7) over β-globin mRNA lacking the BRSK1 3′UTR (compare upper to lower bands; β-globin mRNA contains a total of three DGGGD sequences). There was minimal background binding of the tested mRNAs to the used affinity beads (lanes 8 and 9).

We next tested whether hnRNP F and TTP/BRF proteins affect the turnover of the β-BRSK1 mRNA by monitoring the effect of siRNA-mediated knock-down of hnRNP F or TTP/BRF proteins in pulse-chase β-BRSK1 mRNA decay assays in HeLa Tet-Off cells. The β-BRSK1 mRNA was stabilized (P<0.01; n = 6) following hnRNP F knockdown, degrading with a half-life of 123±12 minutes as compared to 77±6 minutes for cells treated with the control siRNA (Figure 3B). Less than 11% of cellular hnRNP F remained in the siRNA-treated cells, whereas hnRNP H levels were unaffected (Figure 3C). In contrast to hnRNP F, depletion of hnRNP H showed only a minor effect on the half-life of the β-BRSK1 mRNA (Figure S1). We were unable to test the effect of co-depletion of hnRNP F and hnRNP H proteins because this double knockdown impaired cell growth (data not shown). Following treatment with a previously characterized siRNA targeting all three TTP/BRF family proteins [37] the β-BRSK1 mRNA was stabilized and degraded with a half-life of 220±43 minutes (Figure 3B). Thus, hnRNP F stimulates the degradation of an exogenous TTP/BRF-target ARE-mRNA containing multiple binding sites for hnRNP F and TTP.

### The stimulation of TTP/BRF-target mRNA degradation by hnRNP F does not correlate with the extent of hnRNP F mRNA binding

**Figure 4:**
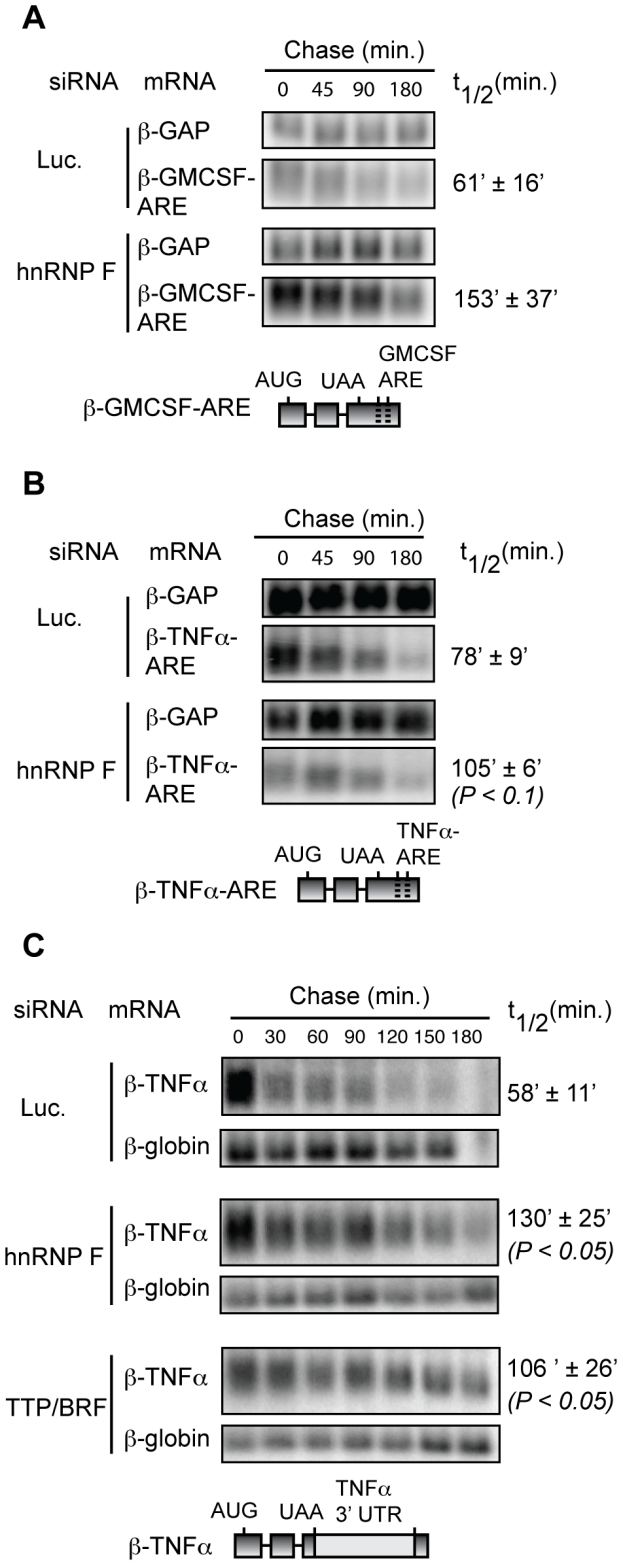
hnRNP F stimulates the decay of TTP/BRF-target mRNAs that show low binding to hnRNP F. (A) Northern blots showing mRNA decay of the β-GMCSF-ARE mRNA reporter in HeLa Tet-off cells transfected with siRNAs targeting hnRNP F or luciferase (Luc.) as indicated. Levels of reporter mRNA was normalized to the constitutively transcribed control mRNA (β-GAP mRNA) and half-life (t_1/2_) and standard error of the mean was determined from two independent experiments. Cartoon of the β-ARE mRNA reporter with the ARE from GMCSF inserted in the 3′ UTR is shown below the panel. (B) Same as panel *A*, but monitoring the decay of a reporter mRNA containing the ARE from the TNFα mRNA. Average half-life and standard error of the mean was calculated from two independent experiments. (C) Same as panel *A*, but monitoring decay of the β-TNFα mRNA reporter, containing the entire 3′ UTR of TNFα mRNA. Average half-life and standard error of the mean was calculated from 4 independent experiments.

Given the observed complex formation of hnRNP F with TTP and BRF1 (Figures 1 and 2), it was possible that the concentration of hnRNP F on mRNAs dictates its ability to stimulate TTP/BRF-mediated mRNA degradation. Alternatively, the relationship could be more complex, depending for example on the position(s) of hnRNP F binding within the mRNA or on other aspects of mRNP composition. We therefore tested whether hnRNP F stimulates the degradation of TTP/BRF-target mRNAs predicted to have less binding to hnRNP F than the BRSK1 3′UTR. We replaced the BRSK1 3′UTR with the AREs from GMCSF and TNFα mRNAs, which are well-characterized targets of TTP [3], [38]–[40], thereby reducing the number of hnRNP F/H consensus sites to only those found in the β-globin mRNA reporter. As expected, these ARE-reporter mRNAs are not enriched over β-globin mRNA lacking an ARE in IPs for hnRNP F (Figure S2 and data not shown). Nevertheless, as seen in Figures 4A and B, β-globin mRNAs containing the AREs from GMCSF (Figure 4A) or TNFα (Figure 4B) are both stabilized following knockdown of hnRNP F. Depletion of hnRNP F also stabilized a β-globin mRNA containing the entire 3′UTR from TNFα mRNA (Figure 4C). This 3′UTR contains nine hnRNP F/H consensus sites, but we detected no enrichment for TNFα mRNA over GAPDH mRNA in hnRNP F/H IP assays from RAW cells (Figure S3). From these experiments we conclude that the ability of hnRNP F to stimulate the decay of TTP/BRF-target mRNAs does not correlate simply with the number of hnRNP F/H consensus binding sites and the extent of hnRNP F mRNA binding, suggesting a more complex mechanism than concentration-dependent recruitment.

### hnRNP F associates with a subset of endogenous TTP-target ARE mRNAs and stimulates LIF mRNA decay in NIH 3T3 cells

**Figure 5:**
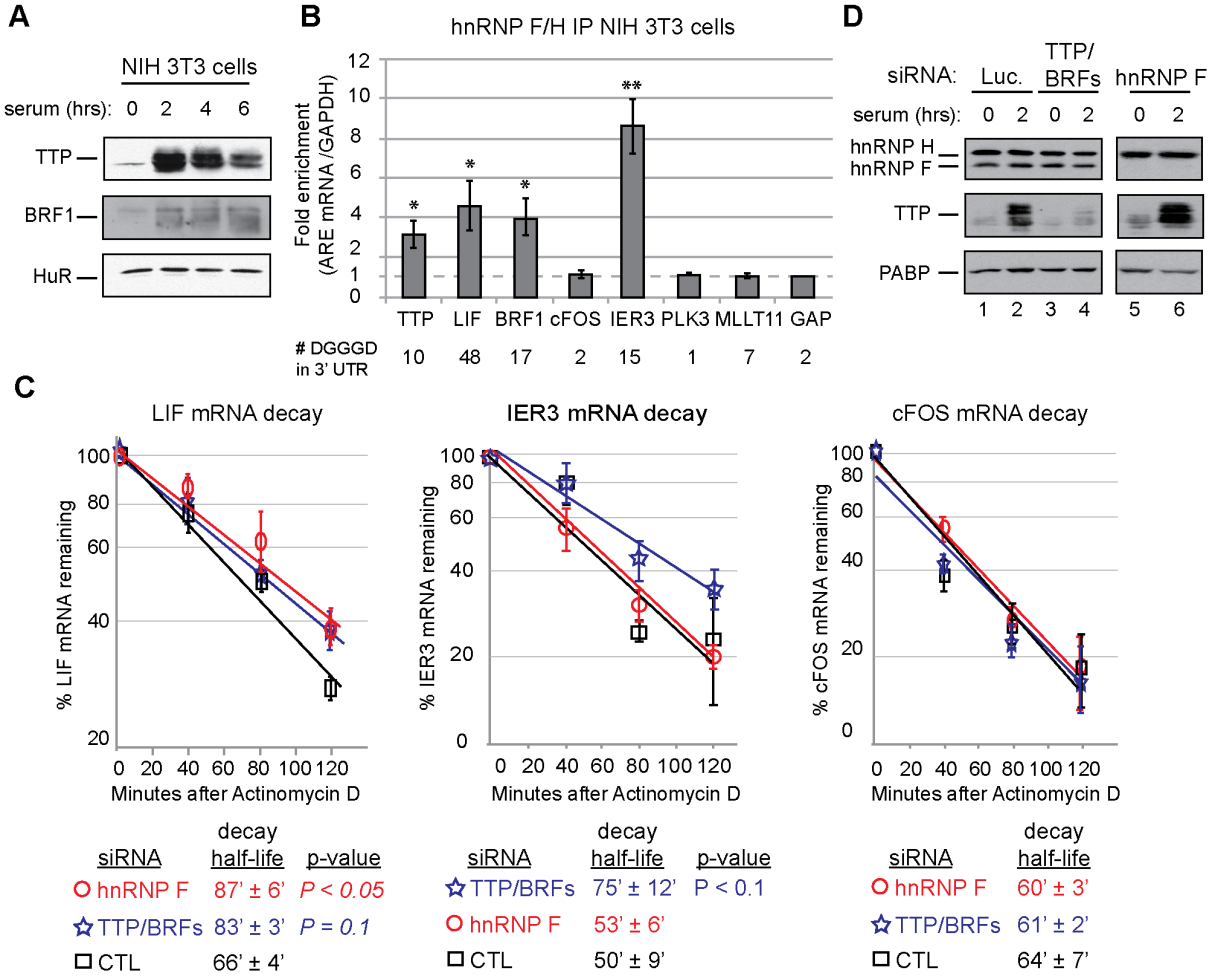
hnRNP F stimulates decay of the endogenous TTP-target LIF mRNA in NIH 3T3 cells. (A) Western blots showing levels of TTP, BRF1, and HuR in NIH 3T3 cells incubated with serum for the indicated time after 24 hours of serum starvation. (B) Quantification of the enrichment relative to GAPDH mRNA of known TTP-target mRNAs (listed below the graph) that co-precipitate with an antibody against hnRNP F/H from extracts of NIH 3T3 cells incubated for 2 hours with serum after 24 hours of serum starvation. mRNA levels were determined by qRT-PCR. The fold enrichment for each ARE-mRNA was calculated by dividing the level of ARE-mRNA relative to that of GAPDH mRNA in each IP sample, after subtracting background levels from IP reactions with anti-Myc. Indicated standard error of the mean values were calculated from three experiments. Asterisks represent P-values <0.1 (*) and P-value <0.05 (**). The number of hnRNP F/H consensus binding sites (DGGGD) within the 3′ UTR of each mRNA is listed below the graph. (C) mRNA decay assays for LIF, cFOS, and IER3 mRNAs in NIH 3T3 cells transfected with 80 nM of the indicated siRNAs and incubated for 3 hours in full media after 24 hours of serum starvation. Samples were collected at 0, 40, 80, and 120 minutes after addition of 10 µg/ml Actinomycin D. ARE mRNA was normalized to GAPDH mRNA by qRT-PCR to determine relative abundance and to calculate mRNA decay half-lives. Average half-life, standard error of the mean and P-values for LIF and cFOS were determined from three biological repeat experiments and two biological repeats for IER3. (D) Western blots showing depletion of hnRNP F and TTP in NIH 3T3 cells transfected with 80 nM of the indicated siRNA and incubated with serum for 0 or 2 hours after 24 hours of serum starvation. PABP serves as a loading control.

We next wished to test whether hnRNP F stimulates degradation of endogenous TTP substrate mRNAs. These experiments were performed in NIH 3T3 fibroblast cells, which express TTP upon stimulation with serum [41]. NIH 3T3 cells were chosen for these experiments because siRNA-mediated knockdowns were more efficient in this cell line than in RAW cells (data not shown). As seen in Figure 5A, TTP and BRF1 proteins are induced in NIH 3T3 cells following serum addition after starvation, with TTP protein levels peaking at 2 to 4 hours and BRF1 induced at 2 to 6 hours.

To first test whether hnRNP F can be found associated with endogenous TTP substrate mRNAs, RNA-IP experiments were performed in NIH 3T3 cells stimulated with serum for two hours. Bound mRNAs were assayed by quantitative (q)RT-PCR and enrichment was determined as the ratio of bound ARE mRNA over GAPDH mRNA, since the co-purification of GAPDH mRNA with the antibody against hnRNP F was the lowest of any of the tested mRNAs (Figure 5B). We specifically monitored for TTP target mRNAs determined from global mRNA decay analyses in TTP^−/−^ mouse embryonic fibroblasts [24] and from TTP IPs [22], [23]. All of the tested ARE mRNAs contain three or more 3′UTR AUUUA sequences and, as expected, were all enriched in TTP IPs over GAPDH mRNA, which contains no AUUUA motifs (Figure S4). As seen in Figure 5B, RNA-IPs from NIH 3T3 cells uncovered a subset of TTP-target mRNAs that were enriched over GAPDH mRNA with the antibody against hnRNP F. These include TTP, LIF, IER3 and BRF1 mRNAs. The hnRNP F antibody does not discriminate between hnRNP F and hnRNP H1/H2 (Figure 2), so it cannot be ruled out that some mRNAs were specifically associated with one or the other of these hnRNPs. By contrast, cFOS, PLK3, and MLLT11 mRNAs were not enriched over GAPDH mRNA with the hnRNP F antibody (Figure 5B). The observed enrichment of individual mRNAs with hnRNP F/H correlates well with the number of hnRNP F/H consensus sites in the mRNA 3′UTRs, with enriched mRNAs containing 10–48 consensus sites compared with 1–7 in those not enriched over GAPDH (Figure 5B, bottom).

Next, decay rates of a subset of the tested mRNAs were measured in NIH 3T3 cells by quantitative (q)RT-PCR at time points following treatment with the global transcription inhibitor Actinomycin D, added three hours after serum stimulation. LIF, IER3, and cFOS mRNAs were monitored in these assays, since these mRNAs reached maximal induction and clearance during this time course (data not shown). LIF mRNA was stabilized (P<0.05) upon hnRNP F knockdown and degraded with a half-life of 87±6 minutes compared to 66±4 minutes for the control siRNA treatment (Figure 5C left panel; knockdown efficiency shown in Figure 5D). LIF mRNA was also stabilized using two other independent hnRNP F siRNAs (Table S1). As expected, knockdown of TTP/BRF proteins also stabilized LIF mRNA, to a half-life of 83±3 minutes (Figure 5C).

In contrast to LIF mRNA, another TTP-target mRNA encoding IER3 did not appear to be stabilized following hnRNP F knockdown (Figure 5C, middle panel) with a measured half-life of 53±6 minutes compared to 50±9 minutes for the control siRNA treatment. As expected, knockdown of TTP/BRF proteins stabilized IER3 mRNA, degrading with a half-life of 75±12 minutes (P<0.1). Thus, hnRNP F appears to be limiting for only a subset of TTP-target mRNAs, and, as also observed with reporter mRNAs in Figure 4, the extent of hnRNP F mRNA association does not correlate with the ability to stimulate decay (compare Figures 5B and 5C). cFOS mRNA (Figure 5C, right panel) served as a negative control for TTP-mediated mRNA decay; although cFOS mRNA contains AREs and is bound by TTP, TTP is not limiting for cFOS mRNA decay [24]. As expected, cFOS mRNA decay was unaffected after TTP/BRF or hnRNP F knockdown. Taken together, we conclude that hnRNP F forms an RNA-independent complex with TTP and BRF1 proteins and stimulates the degradation of a subset of TTP/BRF-target mRNAs.

## Discussion

The regulation of ARE-mRNAs by TTP and its family members is complex and not well understood. For example, much remains unknown about how TTP and BRF1 identify and bind to substrate mRNAs, and how their RNA decay activity is executed and regulated. To address these questions we sought to identify proteins that interact with TTP and BRF1 and might serve as TTP/BRF1 co-factors. Two proteins, hnRNP F and CAD, were identified to complex at high levels with TTP and BRF1 in a RNA-independent manner (Figures 1 and 2). Given its previously established role in mRNA regulatory events we focused our study on hnRNP F and found that it stimulates the degradation of a subset of TTP/BRF1-target mRNAs (Figures 3–5). Stimulation of TTP/BRF1-target mRNA decay did not correlate with the extent of hnRNP F mRNA binding (Figures 3–5), indicating a more complex mechanism than simple concentration-dependent recruitment. Taken together, our observations identify a new component of TTP/BRF-complexes, which serves as a co-factor in a subset of TTP/BRF-mediated decay events.

The specific mechanism by which hnRNP F stimulates TTP/BRF1-mediated decay, and how it interfaces with other TTP/BRF co-factors, is an important question for future study. Given that hnRNP F is a sequence-specific member of the hnRNP class of RNA-binding proteins that are abundant components of mRNPs, an interesting possibility is that TTP/BRF mRNA-target specificity is dictated in part by the hnRNP composition of the mRNP. If true, this could help explain why TTP/BRF proteins act on only a subset of ARE-containing mRNAs [24]. Our observations showed no correlation between the ability of hnRNP F to stimulate ARE-mRNA decay and the number of predicted hnRNP F binding sites or the extent of hnRNP F mRNA binding (Figures 3–5). Thus, the mechanism by which hnRNP F stimulates TTP/BRF-mediated degradation is likely more complex, and could depend for example on the position of hnRNP F binding within the mRNA or other aspects of mRNP structure or composition. Future studies should reveal whether the position of hnRNP F binding relative to the ARE or other mRNA elements is important, or whether additional mRNP components facilitate the communication between hnRNP F and TTP/BRF proteins.

Given that the hnRNP F-TTP/BRF complex is resistant to RNase, it is also possible that hnRNP F stimulates TTP/BRF proteins through mechanisms that do not require direct RNA binding by hnRNP F. hnRNP F could stimulate TTP/BRF activity as part of the TTP/BRF complex for example by affecting the ability of TTP/BRF proteins to recruit mRNA decay factors, to remodel the mRNP in preparation for mRNA degradation, or by influencing TTP/BRF regulation by phosphorylation. We observed a consistent - albeit moderate - reduction in the fraction of cellular TTP that complexes with hnRNP F over time of stimulation of RAW macrophages with LPS (Figure 2). Given that TTP is regulated by phosphorylation and dephosphorylation events during a time course of LPS stimulation, this reduction in hnRNP F association with TTP could signify that the interaction is modulated by phosphorylation. Alternatively, the dramatic increase in TTP levels that occurs during LPS stimulation could render the cellular levels of hnRNP F limiting for the interaction. The remodeling of mRNPs that takes place to allow mRNA degradation is generally associated with repression of translation initiation [46] and hnRNP F has previously been implicated in translational repression [33], [34], [47]; thus, an important question for future study is whether this activity of hnRNP F plays a role in TTP/BRF-mediated degradation of ARE-mRNAs.

Finally, hnRNP F and TTP/BRF proteins are present at high levels in the nucleus of some cell types [42]–[45], and hnRNP F has been shown to modulate pre-mRNA splicing and polyadenylation [31], [32]. Thus, the effect of hnRNP F on TTP/BRF function could be mediated by a nuclear interaction. Such an interaction could affect mRNA degradation by TTP/BRF proteins specifically in the nucleus, or affect the downstream activity of TTP/BRF proteins in the cytoplasm. Future studies should reveal the dynamics of the TTP/BRF-hnRNP F interaction and how it relates with other TTP/BRF co-factors. Here we focused on the most abundant TTP/BRF-associated proteins that could be observed by silver staining of RNase-treated TTP/BRF complexes; however, TTP/BRF proteins also assemble with less abundant factors, including many factors involved in translation repression and mRNA degradation [4]–[7]. An important goal of future study is to dissect the dynamics of the TTP/BRF complexes to understand how they control the translation and degradation of a specific subset of ARE-mRNAs in a manner regulated by phosphorylation events.

## Materials and Methods

### Protein isolation and mass spectrometry

The preparation of stable T-REx HEK 293 cell lines expressing FLAG-tagged TTP or BRF1 under control of a tetracycline regulated promoter, and subsequent protein expression, isolation and mass spectrometry were conducted as described previously for decapping factors [6].

### Co-immunoprecipitation assays

RAW264.7 cells seeded in 15 cm plates were cultured in DMEM/10% fetal bovine serum/1% penicillin and streptomycin. 100 ng/ml LPS (Sigma) was added to cells for 0, 2, or 6 hr and cells were washed in PBS, pelleted, and lysed in 1.4 ml lysis buffer (10 mM Tris-HCl pH 7.5, 10 mM NaCl, 2 mM EDTA, 0.1% Triton X-100, 1 mM PMSF, 2 µg/ml aprotinin, 2 µg/ml leupeptin) and incubated on ice for 5 min. NaCl was added to 150 mM and 15 µl RNase A (10 mg/ml) was added and incubated on ice for 10 min. Cytoplasmic extract was collected after a 15 min spin at 14,000 rpm at 4°C and incubated with 80 µg of Protein A sepharose (GE Healthcare Life Sciences, 17-0780-01), in Net-2 buffer (50 mM Tris-HCl, 150 mM NaCl, 0.05% Triton X-100) conjugated to 4 µL of anti-TTP antibody (Sigma, T5327) or pre-immune rabbit serum. For hnRNP F IPs, an equivalent amount of Protein G sepharose was used with 4 µl anti-hnRNP F/H antibody (Abcam, 1G11) or anti-Myc antibody (Cell Signaling, 9B11). After 3 hrs rotating incubation at 4°C, the precipitates were washed 8 times in Net-2 buffer and eluted in 50 ul of load buffer (100 mM Tris-HCl pH 6.8, 4% SDS, 0.2% bromophenol blue, and 20% glycerol) to which 200 mM DTT was added. Precipitated protein was analyzed by SDS-PAGE and Western blotting.

### Pulse chase mRNA decay assays

mRNA decay assays and Northern blotting were performed as previously described [48]. HeLa Tet-off cells (Clontech) were seeded to 12-well plates and cultured in DMEM/10% fetal bovine serum/1% penicillin and streptomycin (full DMEM). siRNAs were transfected at a final concentration of 20 nM using siLentFect Lipid Reagent (Bio-Rad) following the manufacturers protocol. Then either: 1) 24 hours later, plasmid DNA was transfected in the presence of 50 ng/ml tetracycline using TransIt HeLa-Monster (Mirus), or 2) 48 hours later 20 nM siRNA was transfected with plasmid DNA using Lipofectamine 2000 reagent (Inivitrogen) in the presence of 50 ng/ml tetracycline. Two days after plasmid transfection, transcription of the mRNA reporter was activated for six hours with a PBS wash and replacement of full DMEM without tetracycline. Six hours later, transcription was shut off with addition of 1 µg/ml tetracycline. Cells were harvested in Trizol (Invitrogen) for RNA extraction at subsequent time points as indicated, with time “0” taken 20 minutes after tetracycline addition. For each time course, one well of cells were taken up in 2x SDS load buffer (100 mM Tris-HCl pH 6.8, 200 mM DTT, 4% SDS, 0.2% bromophenol blue, and 20% glycerol) for protein analysis by SDS-PAGE and Western blot.

### Endogenous NIH 3T3 cell mRNA decay assays

NIH 3T3 cells were seeded at ∼20% confluency in 10 cm plates in full DMEM. 80 nM siRNA was transfected with TransIT TKO (Mirus) reagent the next day. 24 hours later, cells were washed 3 times with PBS and trypsinized and re-plated at ∼25% confluency in DMEM/0.2% fetal bovine serum/1% penicillin and streptomycin. 24–48 hours later cells were re-fed with DMEM/20% fetal bovine serum/1% penicillin and streptomycin for 3 hrs and then 10 ug/ml Actinomycin D was added and cells were collected at the indicated time points in Trizol (Invitrogen) for RNA extraction.

### RNA immunoprecipitation (RNA-IP) assays

NIH 3T3 cells were seeded at ∼20% confluency in 10 cm plates in full DMEM. The following day cells were washed 3 times with PBS and cultured in DMEM/0.2% fetal bovine serum/1% penicillin and streptomycin. 24–48 hours later cells were re-fed with DMEM/20% fetal bovine serum/1% penicillin and streptomycin. Cells were collected for RNA-IP two hours later as described for protein immunoprecipitation except that the lysis buffer was supplemented with 14 µl yeast total RNA at 10 mg/ml, 1.4 µl RNaseOUT, and NaCl to 150 mM, and that RNase A was not added to extracts. Immunoprecipitates were harvested in Trizol (Invitrogen) for RNA extraction.

For RNA-IP assays from transiently transfected HEK 293T cells, these were seeded at ∼20% confluency on 10 cm plates in full DMEM and transfected the following day with TransIT 293 transfection reagent (Mirus) and 5 µg pcDNA3-βwtβ-BRSK1-3′UTR, 1 µg pcDNA3-βwtβ mRNA reporter plasmid and either 4 µg pcDNA3-Flag-hnRNP F, or 500 ng pcDNA3-Flag-TTP and 3.5 µg pcDNA3-Flag plasmid. Two days later cell extracts were collected for RNA-IP as described above for NIH 3T3 cells.

### Quantitative RT-PCR and oligos

3 ug total RNA was treated with DNase I (Invitrogen) and reverse transcribed with either SuperScript II or III (Invitrogen) following the manufacturers protocol. cDNA was used for qPCR with SYBR Green PCR Master Mix (Applied Biosystems) and run on an Applied Biosystems StepOnePlus Real-Time PCR System. Gene expression levels were quantified relative to endogenous GAPDH. P-values were calculated using Student's t-test (paired two tailed). The following oligos were used: TTP-F - cggaggactttggaacataaac, TTP-R - ggagttgcagtaggcgaagtag, GAPDH-F - catggccttccgtgttccta, GAPDH-R - cctgcttcaccaccttcttgat, LIF-F- tgtgcaacaagtaccgtgtg, LIF-R- ttgcttgtatgtccccagaag, TNFA-F - accttgtctactcccaggttctc, TNFA-R - gaggttgactttctcctggtatg, IL10-F - tgctatgctgcctgctcttac, IL10-R – aagtgggtgcagttattgtcttc, USP46-F - tgcttcaagcgctgtacttc, USP46-R - tgacgccaaccttcttcttc, KLHL2-F - accaaaggctatccgaagtg, KLHL2-R – aagccaccaacagcaaagac, IER3-F - gcgcgtttgaacacttctc, IER3-R- cagaagatgatggcgaacag, cFOS-F - gaatggtgaagaccgtgtcag, cFOS-R - gtctccgcttggagtgtatc, PLK3-F - ttgcgtcctacatggaacag, PLK3-R - actgaggatcagcttcgtgtg, MLLT11-F - tattgccagcatccactctg, MLLT11-R - cagcaccaccagcacaatag.

### siRNAs

siRNAs were purchased from Dharmacon. Luciferase control siRNA: 5′-CGU ACG CGG AAU ACU UCG AUU-3′+5′-UCG AAG UAU UCC GCG UAC GUU-3′. mTTP siRNA: 5′-GGA GGA CUU UGG AAC AUA AUU -3′+5′- UUA UGU UCC AAA GUC CUC CUU-3′. mBRF1/2 (A) - 5′- UGC CGC ACC UUC CAC ACC ACA UU-3′+5′- UGU GGU GUG GAA GGU GCG GCA UU -3. mBRF1/2 (B) - 5′- CUA CAA GAC GGA GCU GUG CCG UU-3′+5′- CGG CAC AGC UCC GUC UUG UAG UU-3. Target sequences for the following siRNAs are: human TTP/BRF1/2 – CGC UGC CAC UUC AUC CAC AAC UU [37], hnRNP F (1) – AGU CAG AAG AUG AUG UAA A, hnRNP F (2) – GGA AUG UAU GAC CAC AGA UUU, hnRNP F (3) – UGA GAA AGC UUU AGG GAA G, hnRNP H – UAA GCA GUA AGC GUA UUU A. siRNAs targeting Upf1 was described previously [49].

### Plasmid constructs

The BRSK1 3′ UTR was PCR amplified from human genomic DNA using the oligos – F - gga gaa GCGGCCGC TCC TGG CCA CCA ACG GGA CC and R - gga gaa TCTAGA CGG AAT CAG AGA CAC GGA CGC AGG. The amplicon was restriction digested and ligated into the pcTET2-βwtβ reporter plasmid previously described [50] using *Not*I and *Xba*I restriction enzymes. The TNFα 3′ UTR was PCR amplified using the oligos, F - gga gaa GCGGCCGC CGA ACA TCC AAC CTT CCC AAA CGC, R - gga gaa CTGCAG GCT CCT CTC CAG CTC TCT CCG, and restriction enzyme cloned into pcTET2-βwtβ plasmid with *Not*I and *Pst*I. The β-GMCSF-ARE and β-TNFα-ARE reporter plasmids were described previously [50]. The pcDNA3-Myc-TTP and pcDNA3-Flag-TTP plasmids were described previously [5]. hnRNP F was PCR amplified and restriction digest inserted into pcDNA3-Myc and pcDNA3-Flag using *Bam*HI and *Not*I restriction sites.

### Antibodies

The following antibodies were used: Rabbit polyclonal anti-PABP (Abcam; ab21060), mouse monoclonal anti-hnRNP F/H (Abcam, 1G11), mouse monoclonal anti-Myc-tag (Cell Signaling, 9B11), Upf1 rabbit polyclonal antisera [48], mouse monoclonal anti-HuR (3A2) [51], and rabbit polyclonal anti-TTP-N-terminal (Sigma, T5327).

## Supporting Information

Figure S1 **hnRNP H knockdown shows only minor effects on the decay of reporter ARE-mRNAs.** (A) Northern blots showing mRNA decay of the β-BRSK1 mRNA reporter in HeLa Tet-off cells transfected with an siRNA targeting hnRNP H1 and H2 (referred to as hnRNP H). Levels of the reporter mRNA was normalized to the constitutively expressed β-globin control mRNA and the half-life (t_1/2_) was calculated. (B) Northern blots showing mRNA decay of the β-TNFα-ARE mRNA reporter in HeLa Tet-off cells transfected with an siRNA targeting hnRNP H proteins. (C) Western blot showing a representative knockdown of hnRNP H in HeLa Tet-off cells. hnRNP F knockdown is also shown. Upf1 serves as a loading control.
(DOCX)

Figure S2 **hnRNP F IP does not detectably enrich β-GMCSF-ARE mRNA reporter over a control mRNA.** Northern blot showing reporter mRNA containing the ARE from GMCSF mRNA, β-GMCSF-ARE, or control (β-GAP mRNA), that co-precipitates with transiently expressed Flag-tagged hnRNP F (lane 3) or TTP (lane 4) in HEK 293T cells. Precipitates and 5% of total extract (lanes 1, 2) were probed for the presence of β-globin mRNAs.
(DOCX)

Figure S3 **hnRNP F/H IP does not enrich TNFα over GAPDH mRNA.** Quantification of the enrichment of TNFα mRNA and LIF mRNA, relative to GAPDH (GAP) mRNA, in immunoprecipitates with antibodies against TTP (left panel) or hnRNP F/H (right panel) from extracts of RAW264.7 cells stimulated with 100 ng/ml LPS for 2 hours. Below the graphs are shown the number of ARE pentamer sequences (AUUUA; left graph) and the number of hnRNP F consensus binding sites (DGGGD; right graph) found within the 3′ UTR of each mRNA. Fold enrichment were determined from three biological repeats; error bars represent standard error of the mean.
(DOCX)

Figure S4 **Association of TTP with NIH 3T3 cell ARE mRNAs.** Quantification of the enrichment relative to GAPDH (GAP) mRNA of known TTP-target mRNAs (listed below the graph) that co-precipitate with an antibody against TTP from extracts of NIH 3T3 cells incubated for 2 hours with serum after 24 hours of serum starvation. mRNA levels were determined by qRT-PCR. The average-fold enrichment for each ARE-mRNA was calculated from two biological repeats by dividing the level of ARE-mRNA relative to that of GAPDH mRNA in each IP sample, after subtracting background levels from IP reactions with rabbit pre-immune serum; error bars represent standard error of the mean. The number of ARE pentamer sequences (AUUUA) within the 3′ UTR of each mRNA is listed below the graph.
(DOCX)

Table S1 **Summary of LIF mRNA decay in NIH 3T3 cells.** The decay of endogenous LIF mRNA in siRNA-treated NIH 3T3 cells is shown in the table. The decay rate after hnRNP F depletion with two additional siRNAs, hnRNP F (2) and hnRNP F (3), is shown. The mRNA decay rate represents the average half-life ± standard error of the mean from three biological repeats (N = 3).
(DOCX)
