# Supplementary material for: hnRNP F Complexes with Tristetraprolin and Stimulates ARE-mRNA Decay
Source: PLoS One. 2014 Jun 30;9(6):e100992. doi: 10.1371/journal.pone.0100992 (PMC4076271; doi:10.1371/journal.pone.0100992)
Supplement: Figure S1 — hnRNP H knockdown shows only minor effects on the decay of reporter ARE-mRNAs. (A) Northern blots showing mRNA decay of the β-BRSK1 mRNA reporter in HeLa Tet-off cells transfected with an siRNA targeting hnRNP H1 and H2 (referred to as hnRNP H). Levels of the reporter mRNA was normalized to the constitutively expressed β-globin control mRNA and the half-life (t1/2) was calculated. (B) Northern blots showing mRNA decay of the β-TNFα-ARE mRNA reporter in HeLa Tet-off cells transfected with an siRNA targeting hnRNP H proteins. (C) Western blot showing a representative knockdown of hnRNP H in HeLa Tet-off cells. hnRNP F knockdown is also shown. Upf1 serves as a loading control. (DOCX) [file pone.0100992.s001.docx]

**
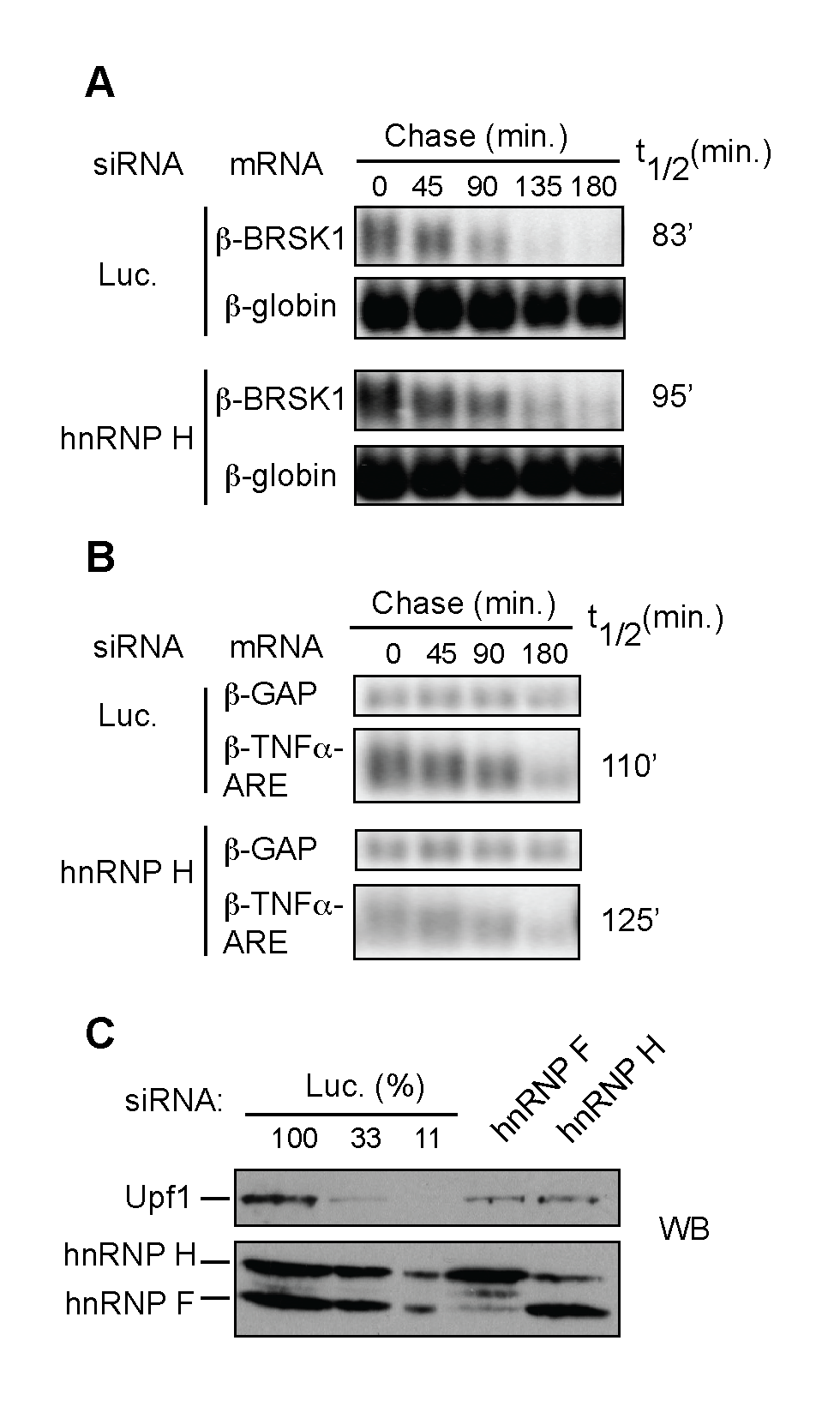
**

**Figure S1. hnRNP H knockdown shows only minor effects on the decay of reporter ARE-mRNAs.**

(A) Northern blots showing mRNA decay of the β-BRSK1 mRNA reporter in HeLa Tet-off cells transfected with an siRNA targeting hnRNP H1 and H2 (referred to as hnRNP H). Levels of the reporter mRNA was normalized to the constitutively expressed β-globin control mRNA and the half-life (t_1/2_) was calculated.

(B) Northern blots showing mRNA decay of the β-TNFα-ARE mRNA reporter in HeLa Tet-off cells transfected with an siRNA targeting hnRNP H proteins.

(C) Western blot showing a representative knockdown of hnRNP H in HeLa Tet-off cells. hnRNP F knockdown is also shown. Upf1 serves as a loading control.
