# Supplementary material for: hnRNP F Complexes with Tristetraprolin and Stimulates ARE-mRNA Decay
Source: PLoS One. 2014 Jun 30;9(6):e100992. doi: 10.1371/journal.pone.0100992 (PMC4076271; doi:10.1371/journal.pone.0100992)
Supplement: Figure S2 — hnRNP F IP does not detectably enrich β-GMCSF-ARE mRNA reporter over a control mRNA. Northern blot showing reporter mRNA containing the ARE from GMCSF mRNA, β-GMCSF-ARE, or control (β-GAP mRNA), that co-precipitates with transiently expressed Flag-tagged hnRNP F (lane 3) or TTP (lane 4) in HEK 293T cells. Precipitates and 5% of total extract (lanes 1, 2) were probed for the presence of β-globin mRNAs. (DOCX) [file pone.0100992.s002.docx]

**Figure S2. hnRNP F IP does not detectably enrich β-GMCSF-ARE mRNA reporter over a control mRNA.**

Northern blot showing reporter mRNA containing the ARE from GMCSF mRNA, β-GMCSF-ARE, or control (β-GAP mRNA), that co-precipitates with transiently expressed Flag-tagged hnRNP F (lane 3) or TTP (lane 4) in HEK 293T cells. Precipitates and 5% of total extract (lanes 1, 2) were probed for the presence of β-globin mRNAs.
