# Supplementary material for: hnRNP F Complexes with Tristetraprolin and Stimulates ARE-mRNA Decay
Source: PLoS One. 2014 Jun 30;9(6):e100992. doi: 10.1371/journal.pone.0100992 (PMC4076271; doi:10.1371/journal.pone.0100992)
Supplement: Figure S3 — hnRNP F/H IP does not enrich TNFα over GAPDH mRNA. Quantification of the enrichment of TNFα mRNA and LIF mRNA, relative to GAPDH (GAP) mRNA, in immunoprecipitates with antibodies against TTP (left panel) or hnRNP F/H (right panel) from extracts of RAW264.7 cells stimulated with 100 ng/ml LPS for 2 hours. Below the graphs are shown the number of ARE pentamer sequences (AUUUA; left graph) and the number of hnRNP F consensus binding sites (DGGGD; right graph) found within the 3′ UTR of each mRNA. Fold enrichment were determined from three biological repeats; error bars represent standard error of the mean. (DOCX) [file pone.0100992.s003.docx]

**
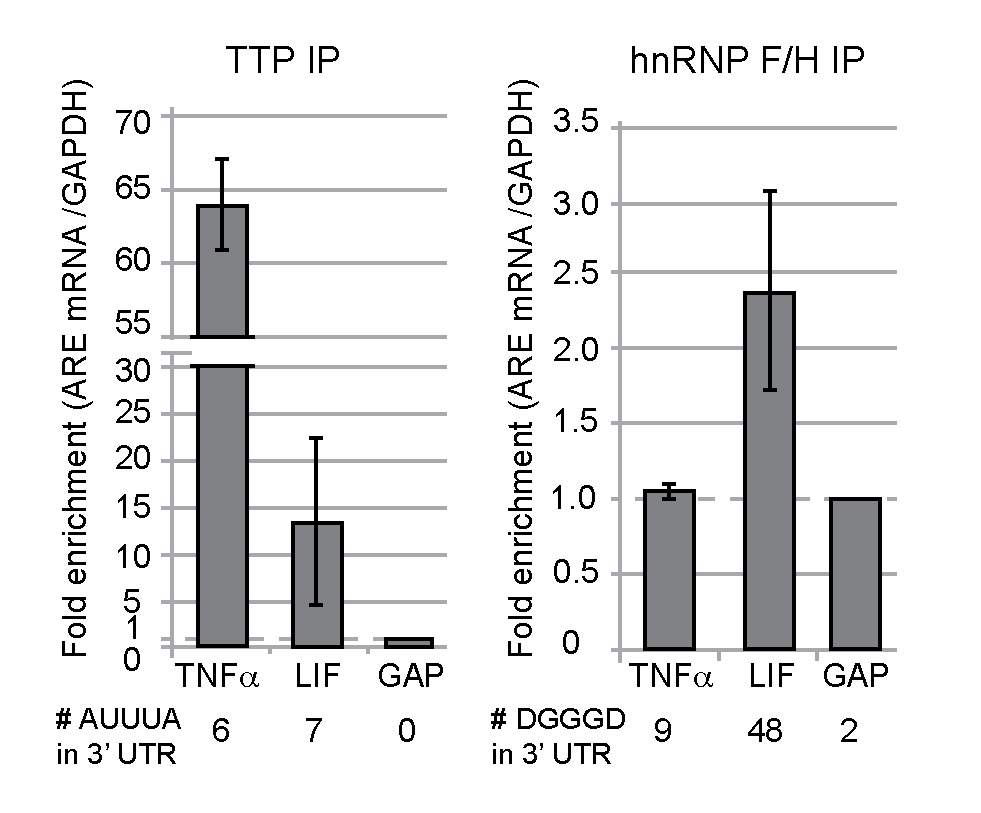
**

**Figure S3. hnRNP F/H IP does not enrich TNFα over GAPDH mRNA.**

Quantification of the enrichment of TNFα mRNA and LIF mRNA, relative to GAPDH (GAP) mRNA, in immunoprecipitates with antibodies against TTP (left panel) or hnRNP F/H (right panel) from extracts of RAW264.7 cells stimulated with 100 ng/ml LPS for 2 hours. Below the graphs are shown the number of ARE pentamer sequences (AUUUA; left graph) and the number of hnRNP F consensus binding sites (DGGGD; right graph) found within the 3’ UTR of each mRNA. Fold enrichment were determined from three biological repeats; error bars represent standard error of the mean.
