# Supplementary material for: hnRNP F Complexes with Tristetraprolin and Stimulates ARE-mRNA Decay
Source: PLoS One. 2014 Jun 30;9(6):e100992. doi: 10.1371/journal.pone.0100992 (PMC4076271; doi:10.1371/journal.pone.0100992)
Supplement: Figure S4 — Association of TTP with NIH 3T3 cell ARE mRNAs. Quantification of the enrichment relative to GAPDH (GAP) mRNA of known TTP-target mRNAs (listed below the graph) that co-precipitate with an antibody against TTP from extracts of NIH 3T3 cells incubated for 2 hours with serum after 24 hours of serum starvation. mRNA levels were determined by qRT-PCR. The average-fold enrichment for each ARE-mRNA was calculated from two biological repeats by dividing the level of ARE-mRNA relative to that of GAPDH mRNA in each IP sample, after subtracting background levels from IP reactions with rabbit pre-immune serum; error bars represent standard error of the mean. The number of ARE pentamer sequences (AUUUA) within the 3′ UTR of each mRNA is listed below the graph. (DOCX) [file pone.0100992.s004.docx]

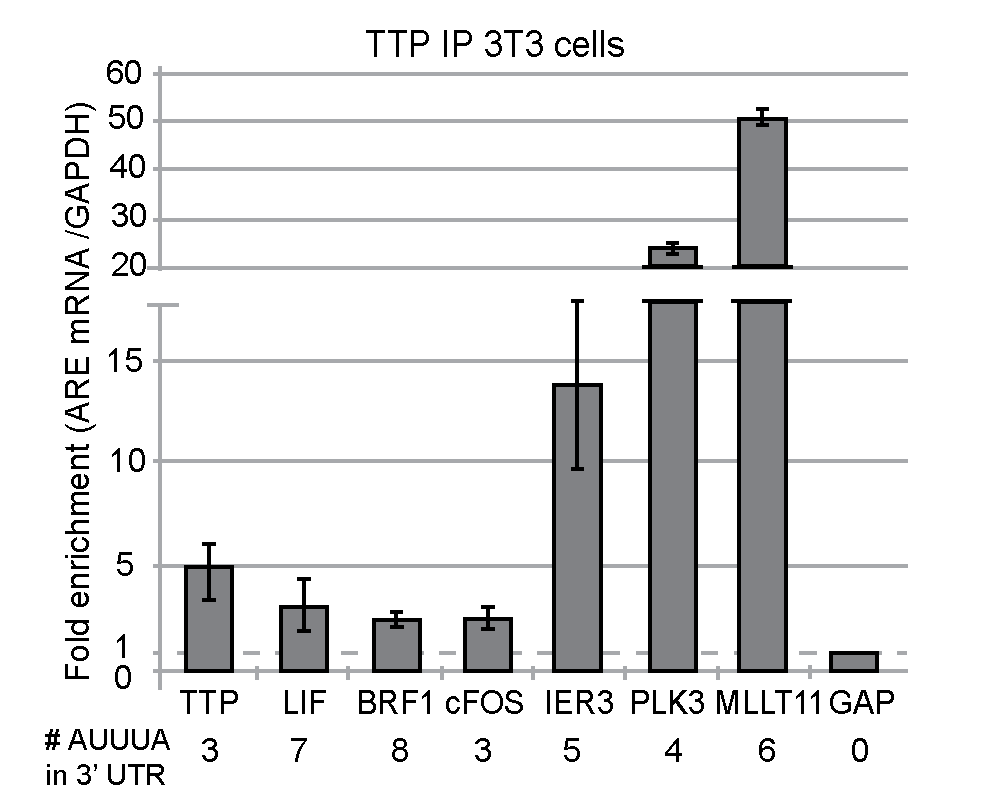


**Figure S4. Association of TTP with NIH 3T3 cell ARE mRNAs.**

Quantification of the enrichment relative to GAPDH (GAP) mRNA of known TTP-target mRNAs (listed below the graph) that co-precipitate with an antibody against TTP from extracts of NIH 3T3 cells incubated for 2 hours with serum after 24 hours of serum starvation. mRNA levels were determined by qRT-PCR. The average-fold enrichment for each ARE-mRNA was calculated from two biological repeats by dividing the level of ARE-mRNA relative to that of GAPDH mRNA in each IP sample, after subtracting background levels from IP reactions with rabbit pre-immune serum; error bars represent standard error of the mean. The number of ARE pentamer sequences (AUUUA) within the 3’ UTR of each mRNA is listed below the graph.
