# Supplementary material for: hnRNP F Complexes with Tristetraprolin and Stimulates ARE-mRNA Decay
Source: PLoS One. 2014 Jun 30;9(6):e100992. doi: 10.1371/journal.pone.0100992 (PMC4076271; doi:10.1371/journal.pone.0100992)
Supplement: Table S1 — Summary of LIF mRNA decay in NIH 3T3 cells. The decay of endogenous LIF mRNA in siRNA-treated NIH 3T3 cells is shown in the table. The decay rate after hnRNP F depletion with two additional siRNAs, hnRNP F (2) and hnRNP F (3), is shown. The mRNA decay rate represents the average half-life ± standard error of the mean from three biological repeats (N = 3). (DOCX) [file pone.0100992.s005.docx]

**Table S1. Summary of LIF mRNA decay in NIH 3T3 cells.**

| siRNA | half-life (min.) | N | P-value |
| --- | --- | --- | --- |
| Luc. | 66’ ± 4’ | 3 | - |
| hnRNP F (1) | 87’ ± 6’ | 3 | P < 0.05 |
| hnRNP F (2) | 78’ ± 9’ | 3 | P = 0.35 |
| hnRNP F (3) | 84’ ± 7’ | 3 | P = 0.1 |
| TTP/BRF | 83’ ± 3’ | 3 | P = 0.1 |

The decay of endogenous LIF mRNA in siRNA-treated NIH 3T3 cells is shown in the table. The decay rate after hnRNP F depletion with two additional siRNAs, hnRNP F (2) and hnRNP F (3), is shown. The mRNA decay rate represents the average half-life ± standard error of the mean from three biological repeats (N=3).
